# Properties of particle phases for metal-matrix-composite design

**DOI:** 10.1016/j.dib.2017.04.038

**Published:** 2017-04-29

**Authors:** C. Baron, H. Springer

**Affiliations:** Max-Planck-Institut für Eisenforschung GmbH, Düsseldorf, Germany

**Keywords:** Metal-matrix-composites, Particles, Properties

## Abstract

Successful metallurgical design of metal-matrix-composites relies on the knowledge of the intrinsic property profiles of the metal matrix and especially the compounds employed for particles, whiskers or fibres. In this work we compiled the key properties melting point, bulk modulus, shear modulus, Young׳s modulus, density, hardness, Poisson׳s ratio and structure/space group from the widespread literature data for the most relevant compound types, i.e. borides, carbo-borides, carbides, oxides, nitrides and intermetallic phases.

**Specifications Table**

**Table:** 

Subject area	*Physical metallurgy, Material Science, Engineering*
More specific subject area	*Metal-matrix-composites*
Type of data	*Table*
How data was acquired	*Literature survey*
Data format	*Raw, processed*
Experimental factors	–
Experimental features	–
Data source location	–
Data accessibility	Data are accessible in this article

**Value of the data**

• The comprehensive data collection allows straightforward comparisons of individual properties, types and groups of compounds.
• Readily obtainable ratios of properties allow judging particles concerning their suitability for specific design goals (such as the material stiffness/density ratio).
• Specific effects of particles on the properties of the bulk composite can be estimated, for example regarding the co-deformation of particles and matrix influenced by their crystallographic coherency, or the amount of particles required for a specific gain in stiffness.

## 1 Data

Metal-matrix-composites allow overcoming the specific limitations of metallic and ceramic materials by blending their typically mutually exclusive property profiles. Knowledge based design of the composites requires, depending on the desired property profile and application, the choice of suitable metallic matrices and particles characterized by their intrinsic properties. In the following table the intrinsic properties (melting point, bulk modulus (*B*), shear modulus (*G*), Young׳s modulus (*E*), density, hardness, Poisson׳s ratio and structure/space group) of different types of phases (borides, carbo-borides, carbides, oxides, nitrides and intermetallics) are compiled from literature sources. The reference for each value or range of values is listed next to it on the right. Unless specified otherwise, values were assumed to have been determined experimentally as specifications are in most cases not given in the listed references. Densities determined by X-ray diffraction (XRD) are enclosed in curved brackets {}. Theoretically determined values are marked with a star *. Furthermore, the main selection criteria brittleness (expressed by the *B*/*G* ratio; *B*/*G* values below 1.75 are considered to represent ‘more brittle’ compounds [1]) and specific modulus (i.e. the *E*/density ratio) have been derived. If more than one value is given for *E* and density of a compound, i.e. several values from one reference or diverging values from different references, the *E*/density ratio is given as a range. In case of several values listed for *B* and *G*, the determined *B*/*G* ratio was chosen conservatively using the lowest *B* and highest *G* value, respectively (Table 1).

**Table 1:** 

														**Legend:**^⁎^theoretical value; {} XRD;			
**Phase**	Melting point / °C	Ref.	**B** Bulk modulus / GPa	**G** Shear modulus / GPa	Ref.	**B / G**	***E*** Young's modulus / GPa	Ref.	Density / g cm^-3^	Ref.	**Specific modulus / GPa cm**^**3**^**g**^**-1**^	Hardness / GPa	Ref.	Poisson's ratio	Ref.	Space group / structure	Ref.
**Borides**																	
**AlB**_**2**_	975	[2]	190^⁎^	95^⁎^	[3]	**2.00**	244.4^⁎^	[3]	{3.19}	[4]	**76.6 - 90.5**	23.6	[4]	0.29^⁎^	[3]	P6/mmm	[3]
									2.9	[5]						P6/mmm	[2]
									2.955	[6]							
									2.7	[7]							
**BeB**_**2**_	>1970	[2]	215^⁎^		[8]				2.32 - 2.48	[2]		31.2	[9]			P6/mmm	[2]
	~ 1700	[6]															
**BeB**_**6**_	~ 2100	[2]							2.35 / {2.33}	[2]		25.3	[9]			P43212	[2]
	2300	[6]															
**CrB**	1515 / 1550+-50	[4]							6.05	[4]		20.9	[4]			Cmcm	[2]
	~ 2060	[2]							6.04 / {6.11}	[2]		19.2 - 22.9	[10]				
	2050	[6]							6.05 / {6.11}			11.8 - 12.7	[6], [9]				
**CrB**_**2**_	2280	[2]	239.2^⁎^	139.9^⁎^	[3]	**1.71**	415.4^⁎^	[3]	[5.6]	[4]	**34.0 - 79.6**	17.7	[4]	0.26^⁎^	[3]	P6/mmm	[2]
	1960 / 1900 / 1850+-50	[4]					211	[4]	5.22 / {5.60}	[6]		20.3 - 22.5	[10]			hex (c-32 type)	[4]
	2200+-50	[6]					210.9	[9]	6.2	[7]		20.6	[6]				
	2100	[7]					235.6	[7]			**~ 38.0**[7]						
**CrB**_**4**_	1400 - 1600	[9]	265^⁎^	261^⁎^	[11]	**1.02**	312^⁎^	[12]				48.0^⁎^	[11]	0.12^⁎^	[11]		
**Cr**_**3**_**B**_**2**_	1960	[4]							6.13 / 6.7	[4]						orthorhombic	[4]
	1960	[7]							6.1	[7]							
**ErB**_**2**_			137.4^⁎^	119.7^⁎^	[3]	**1.15**	278.3^⁎^	[3]	-		**41.5**			0.16^⁎^	[3]	P6/mmm	[2]
**Fe**_**2**_**B**	1389	[13]	249.7^⁎^	60.2^⁎^	[14]	**4.15**	290	[13]	7.15	[13]	**25.7 - 41.4**	13.1+-0.5	[9]			I4/mcm	[2]
	1389	[2]	194^⁎^	67^⁎^	[14]	**2.90**	190^⁎^	[14]	~ 7.0	[2]							
	~ 1390	[4]	331^⁎^	152.8^⁎^	[14]	**2.17**	184^⁎^	[14]									
	1410	[9]					284.4	[9]									
**FeB**_**4**_			253^⁎^	177^⁎^	[11]	**1.43**	475.71^⁎^	[15]				24.2^⁎^	[11]	0.2^⁎^	[15]		
			264.73^⁎^	197.97^⁎^	[15]												
**GdB**_**2**_			131.2^⁎^	113.5^⁎^	[11]	**1.16**	264.3^⁎^	[3]						0.16^⁎^	[3]	P6/mmm	[2]
**HfB**_**2**_	3100 / 3060 - 3065 / 3250 +-100	[4]	260.9^⁎^	233^⁎^	[3]	**1.12**	538.7^⁎^	[3]	11.2	[4]	**45.9 - 58.5**	28.4+-4.9	[9]	0.16^⁎^	[3]	hex (c-32 type)	[4]
	3250	[9]	215	233	[16]	**0.92**	514	[16]	11.19	[17]		28.0^⁎^	[16]	0.12	[16]	hex	[17]
	3380	[17]	265 - 288^⁎^	240 - 273^⁎^	[16]	**0.97 - 1.2**	554 - 614^⁎^	[16]	10.5 / {11.2}	[2], [6]		21.2 - 28.4	[17]	0.124 - 0.159^⁎^	[16]	P6/mmm	[2]
	3250	[2]					530	[17]				28.4	[6]	0.12	[17]		
	3250+-100	[6]															
	3250	[7]									**~ 49.0**[7]						
**LaB**_**6**_	> 2100	[4]					478.6	[9]	{4.72}	[4]	**100.5 - 101.5**	27.2	[9]			Pm3m	[2]
	2530	[2]							4.714	[2]							
									4.76 / {4.72}	[6]							
**LuB**_**2**_			178.4^⁎^	173.3^⁎^	[3]	**1.03**	392.7^⁎^	[3]	{9.656}	[9]	**40.2 - 40.7**			0.13^⁎^	[3]	P6/mmm	[2]
									{9.76}	[6]							
**MgB**_**2**_			151.5^⁎^	116.4^⁎^	[3]	**1.30**	278^⁎^	[3]	2.48 - 2.67 / {2.63}	[9]	**105.5 - 112.1**			0.19^⁎^	[3]		
			120		[8]				2.62 / {2.633}	[2]							
			151		[8]												
**MnB**_**2**_	1988	[9]	220.1^⁎^	121.6^⁎^	[3]	**1.81**	318.4^⁎^	[3]	{5.344}	[9]	**59.3 - 59.6**	16.7+-0.5	[9]	0.31^⁎^	[3]	P6/mmm	[2]
									{5.37}	[6]							
**MnB**_**4**_	2160	[9]	270^⁎^	245^⁎^	[11]	**1.10**						41.5^⁎^	[11]				
												35.3+-1	[9]				
**MoB**_**2**_	2100 / 2250+-50	[4]	302.5^⁎^	186^⁎^	[3]	**1.63**	463.1^⁎^	[3]	{7.78}	[6]	**59.4 - 59.5**	11.7 / 12.6 / 13.5	[4]	0.24^⁎^	[3]	hex. AlB2 structure	[4]
	2350	[9]							{7.78} / {7.8}	[4]						P6/mmm	[2]
**MoB**_**4**_	< ~ 1600	[2]	287^⁎^	239^⁎^	[11]	**1.20**			4.8 / {4.96}	[2], [6]		36.7^⁎^	[11]			P6/mmm	[2]
**Mo**_**2**_**B**	2000 / 2165	[4]							{9.3} / {9.31} / 9.26	[4]		16.3	[4]			CuAl2 structure; c-16 type	[4]
									9.1 / {9.31}	[6]		24.5	[6], [9]				
**Mo**_**2**_**B**_**5**_	decompose to MoB2 @ 1600 - 1650	[4]					671.8	[9]	7.01 / {7.48}	[2], [4], [6]	**89.8 - 95.8**	23.0	[9]			rhombohedral	[4]
	< ~ 1600	[2]														R3m	[2]
**NbB**_**2**_	2900	[4]	286.3^⁎^	210.4^⁎^	[3]	**1.36**	507^⁎^	[3]	6.4 / 6.5 / 6.6 / 6.97 / {7.21}	[4]	**70.4 - 105.8**	16.7 / 25.4	[4]	0.2^⁎^	[3]	hex (c-32 type)	[4]
	~ 3000	[2]					637.5	[9]	6.6	[2]		25.5	[9]			P6/mmm	[2]
	3000	[6]					676.8	[7]	6.97 / {7.00}	[9]							
	3000	[7]							7.2	[7]							
											**~ 94.0**[7]						
**NbB**_**4**_			243^⁎^	194^⁎^	[11]	**1.25**						30.4^⁎^	[11]			P4/mbm	[2]
**NiB**	990	[2]							7.13	[2]		15.2	[9]			Cmcm	[2]
									6.5 / {7.13}	[6]							
**NpB**_**2**_			206.7^⁎^	169.8^⁎^	[3]	**1.22**	399.3^⁎^	[3]	-					0.19^⁎^	[3]		
**PrB**_**6**_	> 2250	[2]							{4.85}	[4]		24.2	[9]				
									4.53 / {4.84}	[2]						Pm3m	[2]
**PuB**_**2**_	1825	[9]	207.4^⁎^	174.1^⁎^	[3]	**1.19**	408.1^⁎^	[3]	{12.674}	[9]	**31.9 - 36.8**	-		0.17^⁎^	[3]		
	< 1200	[2]							{11.1}	[2]						P6/mmm	[2]
									{12.81}	[6]							
**ScB**_**2**_	2250	[2]	243.8^⁎^	256.6^⁎^	[3]	**0.95**	431^⁎^	[3]	3.65 / {3.667}	[9]	**117.4 - 118.1**	17.5+-2.7	[9]	0.11^⁎^	[3]		
	2250	[6]							{3.67}	[2]						P6/mmm	[2]
**SmB**_**2**_			128^⁎^	110.8^⁎^	[3]	**1.16**	258^⁎^	[3]						0.16^⁎^	[3]		
**TaB**_**2**_	3000 - 3150	[4]	295.8^⁎^	191.5^⁎^	[3]	**1.54**	370	[4]	11.7 / {12.6}	[4], [6]	**20.4 - 40.4**	24.9 / 25.6+-1.2 / 16.7	[4]	0.23^⁎^	[3]	hex (c-32 type)	[4]
	3037	[9]					472.5^⁎^	[3]	12.38 / {12.62}	[9]		19 - 25	[17]			hex	[17]
	3040	[17]					265.9	[9]	12.54	[17]		24.5+-0.4	[9]			P6/mmm	[2]
							257	[17]									
**TbB**_**2**_			131.3^⁎^	115.4^⁎^	[3]	**1.14**	267.8^⁎^	[3]	-					0.16^⁎^	[3]	P6/mmm	[2]
**TiB**									5.09 / {4.565}	[9]		26.5- 27.5	[9]			fcc	[4]
									5.09 / 5.26	[2]						F43m	[2]
**TiB**_**2**_	3225	[18]	240	255	[19]	**0.94**	565	[19]	4.52	[18]	**81.0 - 129.9**	25.0	[19]	0.108	[19]	hex P6/mmm	[19]
	2980 / 2900+-80	[4]	250.3^⁎^	260.7^⁎^	[3]	**0.96**	530	[13]	4.5	[19]		25 - 35	[18]	0.11^⁎^	[3]	hex	[18]
	2900	[20]	238	240.4	[21]	**0.99**	366	[4]	4.5	[13]		33.3 / 33.0 / 26.6 / 25.3	[4]	0.109 / 0.11	[22]	hex (c-32 type)	[4]
	2790	[9]					581^⁎^	[3]	4.5 / 4.52 / {4.52}	[4]		25 - 33	[17]			P6/mmm	[2]
	3225	[17]					594^⁎^ / 569	[22]	4.5	[20]		33.0+-0.6	[9]			hex	[17]
	2800	[2]					370	[20]	4.52	[17]						hex	[20]
	2980	[7]					529.6	[9]	4.38	[2]							
							551	[17]	4.5 / {4.52}	[6]				0.11	[17]		
							529.6	[6]	4.5	[7]							
							535.5	[7]			**119**[7]						
**TiB**_**4**_			226^⁎^	190^⁎^	[11]	**1.19**						32.2^⁎^	[11]				
**TmB**_**2**_			137.5^⁎^	120.5^⁎^	[3]	**1.14**	279.7^⁎^	[3]						0.16^⁎^	[3]		
**UB**_**2**_	2385	[2]	205.5^⁎^	209.5^⁎^	[3]	**0.98**	469.6	[3]	{12.692}	[9]	**36.9 - 37.0**	13.6	[4]	0.12^⁎^	[3]	P6/mmm	[2]
									{12.71}	[4]		14.8	[9]				
**VB**	~ 2250	[2]							5.44 / {5.28}	[4]						orthorhombic / Cmcm	[2], [4]
									{5.44}	[6]							
**VB**_**2**_	2040 - 2160	[4]	279.5^⁎^	240.9^⁎^	[3]	**1.16**	562.2^⁎^	[3]	4.61 / 5.28 / {5.10}	[4]	**50.7 - 122.0**	20.4	[4]	0.16^⁎^	[3]	hex (c-32 type)	[4]
	~ 2400	[2]					267.7	[9]	5.06 - 5.28	[9]		27.5+-0.1	[9]			P6/mmm	[2]
	2400+-50	[6]							4.61 / {5.10}	[2]							
	2100	[7]							5.28 / {5.10}	[6]							
									5.1	[7]							
**VB**_**4**_			241^⁎^	237^⁎^	[11]	**1.02**						45.2^⁎^	[11]				
**W**_**2**_**B**	2770+-80	[4]	322.5^⁎^	164.1^⁎^	[3]	**1.97**	420.9^⁎^	[3]	16 / {10.72}	[6]	**24.5 - 39.3**	23.5	[6]	0.28^⁎^	[3]	tetragonal	[4]
									17.17 / 16 / 15.98 / {16.72}	[4]							
**YB**_**2**_	2100	[2]	173.5^⁎^	145.3^⁎^	[3]	**1.19**	340.8^⁎^	[3]	{3.370}	[9]	**101.1 - 117.1**			0.17^⁎^	[3]	P6/mmm	[2]
									{2.91}	[6]							
**YbB**_**2**_			153.7^⁎^	130.2^⁎^	[3]	**1.18**	304.6^⁎^	[3]						0.17^⁎^	[3]		
**YbB**_**6**_	1538 +-33	[4]							5.45 / {5.56}	[4]		25.5	[9]			Pm3m	[2]
	> 2000	[9]															
**ZrB**	2922 / 2992 +-50	[4]							5.7 / {6.7}	[2], [4], [6]		69 - 72 HRA	[4]			cubic / Fm3m	[2], [4]
												35 - 36	[9]				
**ZrB**_**2**_	3000	[18]	220	225	[16]	**0.98**	350	[18]	6.1	[18]	**55.6 - 104.8**	22 - 26	[18]	0.13^⁎^	[3]	hex	[18]
	2980 / 2990+-50 / 3040+-50	[4]	245	243	[16]	**1.01**	498 - 638	[4]	{6.09} / {6.102}	[4]		23+-0.9	[16]	0.144	[4]	hex (c-32 type)	[4]
	2990	[20]	238.6^⁎^	231.4^⁎^	[3]	**1.03**	524.6^⁎^	[3]	6.1	[20]		15.3 / 21.6 / 87 - 89 HRA	[4]	0.14	[22]	hex	[17]
	3200	[9]	238^⁎^ - 276^⁎^	239^⁎^ - 260^⁎^	[16]	**0.92 - 1.15**	343.2	[9]	6.1	[17]		25.3 - 28.0	[17]	0.09 - 0.28	[22]	P6/mmm	[2]
	3245	[17]	207.6	192.2	[4]		554 / 502	[16]	6.17 / {6.09}	[2]		22.1	[6]	0. 109 / 0.13	[16]		
	3040	[2]					520^⁎^ - 555^⁎^	[16]	6.1	[7]		22.1+-0.2	[9]	0.137 - 0.144^⁎^	[16]		
	3060	[7]					350	[20]									
							500	[17]						0.11	[17]		
							518.5	[7]			**~ 85**[7]						
**ZrB**_**4**_			199^⁎^		[11]												
																
**Carbo-Borides**																	
**Hf**_**2**_**BC**			207	150	[23]	**1.38**	362^⁎^	[23]									
**Mo**_**2**_**BC**	2800	[2]	313	181	[23]	**1.73**	466 - 473	[21]	8.71	[2]	**53.5 - 54.3**			0.26	[21]	Cmcm	[2]
			324	185 - 188	[21]	**1.72 - 1.75**											
**Nb**_**2**_**BC**			259	163	[23]	**1.59**	404^⁎^	[23]									
**Ta**_**2**_**BC**			286	168	[23]	**1.70**	421^⁎^	[23]									
**Ti**_**2**_**BC**			208	158	[23]	**1.32**	378^⁎^	[23]									
**V**_**2**_**BC**			260	178	[23]	**1.46**	435^⁎^	[23]									
**W**_**2**_**BC**			350	184	[23]	**1.90**	468^⁎^	[23]									
**Zr**_**2**_**BC**			187	128	[23]	**1.46**	312^⁎^	[23]									
**Carbides**																	
**B**_**4**_**C**	2350	[13]	247^⁎^	200^⁎^	[11]	**1.24**	448	[13]	2.52	[18]	**177.8 - 191.1**	37 - 47	[18]			orthorhombic	[18]
	2450	[18]	175 (graph)		[24]		450	[18]	2.52	[20]		30 (graph)	[24]			orthorombic	[20]
	2450	[20]					472^⁎^	[11]	2.51 / 2.484 / 2.47	[2]		31.7^⁎^	[11]			R3m	[2]
	2450	[2]					450	[20]									
	2420	[7]															
**Cr**_**3**_**C**_**2**_	1800	[13]					371	[13]	6.74	[13]	**55.0 - 55.8**	17.7	[9]			orthorhombic	[4]
	1830 - 1890	[4]					370 @ 449 °C	[25]	6.68 - 6.7	[4]						Pnma	[2]
	1895	[9]					372.7	[9]									
	~ 1900	[2]															
	1985	[7]															
**Cr4C**	1510	[7]															
**Cr**_**7**_**C**_**3**_	1782	[2]	311.7^⁎^ / 309^⁎^	143.9^⁎^	[14]	**2.15**	371 / 374	[14]	6.9	[2]	**53.8 - 54.2**	18.5	[9]			P31c	[2]
	1780	[7]															
**Fe**_**3**_**C**			259.2^⁎^	119.6^⁎^	[14]	**2.17**	177	[26]	7.4	[4]	**23.9**			0.26	[26]		
**HfC**	3000–3900	[13]					317	[13]	12.2	[13]	**24.8 - 23.8**	24.8 - 31.4	[9]				[13]
	3890	[20]					400	[20]	12.3	[20]		26.0	[17]			cubic	[20]
	3900	[17]					352.1	[9]	12.76	[17]		29.0+-3.0	[27]			fcc	[17]
	3890	[2]					352	[17]								Fm3m	[2]
**Mo**_**2**_**C**	2410	[28]					228	[13]	8.9	[13]	**24.8 - 59.9**	14.7+-1.3	[9]			P63/mmc	[2]
	2522	[2]					530 @ 390 °C	[25]	9.04 / {9.18}	[9]							
							533.5	[9]									
**NbC**	1900	[13]					338	[13]	7.6	[13]	**43.2 - 72.7**	23.0+-3.0	[27]			B1 fm3m	[29]
	3613	[9]					492.93 - 549.66	[30]	7.56 / {7.82}	[9]						Fm3m	[2]
	3600	[2]					338.3	[9]	7.8	[7]							
	3775	[7]					540 @ 474 °C	[25]									
							350 - 500	[31]									
							546	[7]			**70.0**[7]						
**Nb**_**2**_**C**	3100	[28]							7.86 / {7.85}	[9]						Pnma	[2]
	3035	[9]							6.7	[7]							
	2675	[7]															
**PKD**			463.1^⁎^ / 442^⁎^	540^⁎^	[32]	**0.82**	1013 ± 52.6	[33]	3.51	[33]	**273.6 - 303.6**	70.0	[32]	0.144 ± 0.055	[33]	cubic	[33]
			368	397.5	[15]		1185.3	[15]						0.0077	[15]		
**SiC**	2200	[18]					480	[18]	3.2	[18]	**117.1 - 158**	20 - 35	[18]	0.17	[34]	hex	[18]
	2300	[20]		200	[34]		480	[20], [34]	3.21	[20]		32.0	[17]	0.17	[35]	hex	[20]
	2820	[17]					386.4	[9]	3.21	[17]				0.16	[17]	polymorphic	[17]
	2750	[7]					427	[35]	3.123 / 3.213	[2]						F43m	[2]
							415	[17]	3.3	[7]							
							495	[7]			**150**[7]						
**TaC**	3880 - 3915	[13]					336	[13]	13.9	[13]	**19.7 - 24.2**	18.2	[17]			Fm3m	[2]
	3880	[20]					290	[20]	14.5	[20]		16.0+-2.0	[27]			cubic	[20]
	3985	[9]					285.4	[9]	14.5	[17]						cubic	[17]
	3800	[17]					285	[17]									
	3780	[2]															
**TiC**	3140	[28]	242.2	188.5	[36]	**1.28**	400	[18]	4.93	[18]	**64.8 - 110.2**	24 - 32	[18]	0.1908	[36]	fcc	[18]
	3067	[18]	240		[37]		462.9	[36]	4.905^⁎^	[36]		31.4	[9]	0.17	[25]	cubic	[20]
	3140	[20]					320	[20]	4.93	[20]		30.0	[17]			cubic	[17]
	3100	[17]					451	[17]	4.94	[17]		32+-2.0	[27]			Fm3m	
	3150	[2]					451.1	[9]	4.2	[7]							
	3250	[7]					450 @ 617 °C	[25]									
							460	[31]									
							420	[7]			**100**[7]						
**UC**	2520	[9]	163.6	82.6	[36]	**1.98**	212.1	[36]	12.97 / {13.63}	[9]	**15.6 - 17.0**	6.9+-1.5	[9]	0.284	[36]		
							220.7	[9]				9.5+-1.0	[27]				
**VC**	2730	[13]					434	[13]	5.77	[13]	**72.6 - 85.1**	27.5 / 20.4 - 24.6 / 20.4 / 92 HRA	[4]	0.32 @ 552 °C	[25]	cubic fm3m	[29]
	2810 - 2865	[4]					421.7	[9]	5.36 - 5.81	[4]						B1 fcc	[4]
	2648	[9]					420 @ 552 °C	[25]	5.1	[7]							
	2750	[7]															
**V**_**2**_**C**	2200	[28]							{5.75}	[9]							
	2187	[9]							5.8	[7]							
	2150	[7]															
**WC**	2800–2860	[13]	577		[38]		669	[13]	15.63	[13]	**32.8 - 47.1**	20 - 24	[18]	0.31 @ 347 °C	[25]	hex	[18]
	2600	[18]					720	[18]	15.7	[18]		21.6	[9]			hex	[4]
	2867+-50 / 2870 / 2900 / 2777 / 2867 / 2627	[4]					519 / 539.8 / 601.2 / 668.2 / 706.7	[4]	15.60 / 15.63 / 15.7 / {15.8}	[4]		17 / 23.5 / 18.3 / 18.4 / 92 HRA	[4]			Fm3m	[2]
	2780	[20]					730	[20]	15.7	[20]						hex	[20]
	2785	[2]					696.3	[9]	15.5 - 15.7 / {15.77}	[9]							
	2777	[7]					700 @ 347 °C	[25]	15.7	[7]							
**ZrC**	3400	[13]	223.1	169.7	[36]	**1.31**	359	[13]	6.73	[13]	**28.3 - 83.8**	25.5 / 27.8 - 34.1 / 21 / 20.5 / 92.5 HRA	[4]	0.197	[36]	B1 fcc	[4]
	3532+-125 / 3532 / 3530 / 3550 / 3540 / 3175	[4]	214.2	124	[9]	**1.31**	406.2	[36]	6.606^⁎^	[36]		27.0	[17]	0.257	[9]	Fm3m	[2]
	3420	[2]					195.1 / 317.8 / 337.8 / 479.9	[4]	6.9 / {6.661. 6.73. 6.70. 6.44}	[4]		30+-3.0	[27]				
	3420	[20]					390	[20]	6.6	[20]						cubic	[20]
	3400	[17]					550 @ 505 °C	[25]	6.56	[17]						fcc	[17]
	3525	[7]					348.1	[9]	6.8	[7]							
							348	[17]									
							408	[7]			**60**[7]						
**Oxides**																	
**Al**_**2**_**0**_**3**_	2045	[13]	264.5	156.6	[39]	**1.69**	379 @ 1090 °C	[13]	3.98	[13]	**99.2 - 104.1**	20.7	[40]	0.27	[41]	hex	[18]
	2046.7+-8	[40]	136.67^⁎^	165	[34]	**0.83**	395.8	[39]	3.97	[40]		18 - 21	[18]	0.13 - 0.45	[40]	hex. cubic. monoclinic	[40]
	2050	[20]	251.2	163.4	[42]	**1.54**	410	[20], [34]	3.94	[39]		23.0	[20]	0.254	[39]		
				124.55 - 347.36	[40]				3.99	[20]				0.23	[34]		
				186.33 (single crystal)	[40]												
**B**_**2**_**O**_**3**_	450	[40]							1.84	[40]							
**BaO**	~ 1923	[40]							5.72	[40]							
**BeO**	2527	[13]	464.29^⁎^		[20]	**3.29**	190 @ 1090 °C	[13]	3.01	[13]	**122.8 - 129.6**	7.8 / 10 / 12.3 / 14.9	[40]	0.36 - 0.38	[40]	hex	[40]
	2570+-30	[40]		95.91 - 100.03	[40]		372	[39]	3.03	[40]							
	2570	[20]		141	[39]		390	[20]	3.01	[20]							
**CaO**	2580	[20]	120	74.05	[43]	**1.62**	181	[43]	3.32	[20]	**53.2 - 54.5**	6.0	[40]	0.22	[43]	cubic	[40]
	2587	[40]		74.0	[40]				3.4	[40]		6.0+-0.8	[27]	0.22	[40]		
	2614	[7]	218		[42]												
**CeO**_**2**_	2000	[13]		62.47	[40]		185	[13]	7.13	[40]	**25.9**						
	2397	[40]							7.13	[13]							
**Ce**_**2**_**O**_**3**_	2142+-30	[40]	109^⁎^	50.8^⁎^	[44]	**2.15**	132		6.9 - 7.0	[40]	**18.9 - 19.1**						
	2210																
**Co**_**2**_**O**_**3**_	894	[40]							5.18	[40]							
**Cr**_**2**_**O**_**3**_	2300	[40]	240		[45]				5.21	[40]		29.1	[40]			hex	[40]
**FeO**	1368	[40]	154		[46]				5.7	[40]		5.4	[40]			hex. cub.	[40]
			162.7		[47]							5.4+-0.5	[27]			fcc	[46]
**Fe**_**2**_**O**_**3**_	1562	[40]	99.6	94.8	[42]	**1.05**	261	[48]	6.51	[40]	**40.1**	10.8 / 5 - 6.8 / 6.8 - 10.9 / 9 / 9.9 / 9 - 10.4 / 3.5 - 3.8	[40]			hex. cub.	[40]
**HfO**_**2**_	2810	[7]							9.68	[7]							
**MgO**	2800+-13	[40]	156.4	124.3	[39]	**1.26**	317 @ 1090 °C	[13]	3.65	[40]	**68.2 - 88.4**	9.1 - 9.3 / 7.5 / 11.2	[40]	0.36	[40]	cubic	[40]
	2800	[7]	156.57^⁎^	130	[34]	**1.20**	249.09	[49]	3.58	[13]		11.0+-1.5	[27]				
			155	130.1	[42]	**1.19**			3.585	[49]							
				77.48 - 113.76	[40]		294.7	[39]	3.506	[39]							
	2840	[20]	162.3		[47]		310	[20], [34]	3.58	[7]				0.17	[34]		
**MgO**_**2**_	2832	[7]															
**MgAl**_**2**_**O**_**3**_	2135	[7]							3.51	[7]							
**MgV**_**2**_**O**_**4**_	2050	[7]															
**MnO**	1785	[40]							5.18	[40]		5.7	[40]			cubic	[40]
												5.7+-0.8	[27]				
**MnO**_**2**_	847	[40]	34.4						5.026	[40]						tetra. rhom.	[40]
**NbO**	1937	[7]															
**NbO**_**2**_	1902	[7]															
**Nb**_**2**_**O**_**3**_	1772	[40]					134.1	[50]									
**Nb**_**2**_**O**_**5**_	1510	[40]							5.98	[40]		7.3	[40]			rhom.	[40]
	1512	[7]															
**NiO**	1957	[40]							7.45	[40]		4.4	[40]			cubic	[40]
**Sc**_**2**_**O**_**3**_	2405	[40]							3.864	[40]						cubic	[40]
**SiO**_**2**_	1600–1725		37.02	31.14	[39]	**1.19**	73	[13]	2.66	[13]	**27.4 - 33.1**			0.17	[40]	**hex -** rhom - tetra - cub	[40]
	1720	[40]	36.98^⁎^		[13], [39]		72.97	[39]	2.32 - 2.651	[40]		7.5 - 12.3	[40]	0.171	[39]		
	1710	[7]							2.203	[39]							
									2.32	[7]							
**Sm**_**2**_**O**_**3**_	~ 2320	[40]	127.99^⁎^	53.1	[39]	**2.41**	139	[39]						0.319	[39]	monoclinic	[40]
**Ta**_**2**_**O**_**5**_	1877	[40]					179.1	[50]	8.73	[40]	**20.5**					rhom.	[40]
	1785	[7]															
**ThO**_**2**_	3300	[40]	69.34^⁎^	98.07 (303 K)	[40]	**0.71**	137.3	[40]	10	[40]	**13.7 - 32.0**	9.7 - 10.9	[40]	0.17	[40]	cubic	[40]
	3220	[20]	178.5	94.2	[39]	**1.89**	240.4	[39]	9.722	[39]							
	3390	[7]					240	[20]	10.05	[20]				0.275	[39]		
							310.4	[7]	9.7	[7]	**32**[7]						
**TiO**	1737	[40]	270		[37]				4.93 - 5.53	[40]		19.6	[40]	0.28		cubic	[40]
												16.0+-3.5	[27]				
**TiO**_**2**_	1855	[40]	210	113.1	[39]	**1.86**	272	[39]	3.84 - 4.24	[40]	**64.2 - 70.8**	6.0 - 10.7	[40]			tetra. rhom.	[40]
	1857	[7]	210.3	113.5	[42]	**1.85**			4.24	[39]							
			215.2	113.54 (single crystal)	[40]	**1.90**											
**Ti**_**2**_**O**_**3**_	2127	[40]	148.9	69.2	[51]	**2.15**	179.8	[51]	5.02	[51]	**35.8**			0.299	[51]	cubic	[51]
																hex	[40]
**UO**_**2**_	2760	[40]					192.9	[39]	10.5	[40]	**17.6 - 18.6**	7.7 - 8.2	[40]	0.302	[39]	cubic	[40]
	2800	[20]	162	74.1	[39]	**2.19**	162.8 - 245.18	[40]	10.37	[39]							
	2875	[7]							10.97	[20]							
**VO**_**2**_	1545	[40]							4.4	[4]						monoclinic	[40]
**V**_**2**_**O**_**3**_	2376	[40]							4.87	[4]						hex	[40]
**WO**_**3**_	1470	[40]							7.16	[4]						tetra. monoclinic	[40]
	1470	[7]															
**Y**_**2**_**0**_**3**_	2450	[20]	148.9+-3	69.2+-2	[51]	**2.15**	179.8+-4.8	[51]	5.02	[51]	**34.1 - 41.0**	6.8	[40]	0.299	[51]	cubic	[40]
			135.7	66.5	[39]	**2.04**	171.5	[39]	5.03	[39]				0.298	[39]		
			141.5^⁎^		[39]		180	[20]	4.5	[20]							
									4.84	[40]							
**ZnO**	1975	[40]	134	44	[39]	**3.05**	199+-2			[40]	**21.2 - 35.9**	1.5 - 3.1	[40]	0.351	[39]	tet - mono @ 1170 °C	[52]
			143.6	45.5	[42]	**3.16**	119	[39]								hex	[40]
				45.5	[40]												
**ZrO**_**2**_	2690	[40]	137.68^⁎^	75	[34]	**1.84**	190	[34]	5.56	[40]	**30.3 - 35.4**	16.6	[40]	0.27	[34]	monoclinic	[40]
	2700	[7]		77.9	[39]		197	[39]	6.27	[40]						cubic	[40]
									5.75	[7]							
**Nitrides**																	
**AlN**	2300	[20]	159.9 - 207	126.4	[53]	**1.64**	350	[20]	3.25	[20]	**90.5 - 110**					hex	[20]
	2375	[7]					350	[9]	3.2	[54]							
							294.2 - 323.6 / 343.7	[7]	3.2	[7]							
							352	[7]			**110**[7]						
**BC**_**2**_**N**			408	445	[11]	**0.92**	980	[24]				65.2		0.096	[24]		[24]
**BN**	2730	[7]							2.1	[7]							
**c-BN**	2973		400	405	[11]	**0.99**	909	[24]	3.45 - 3.48	[33]	**261.2 - 263.5**	48^⁎^	[11]	0.121	[55]	cub (hex)	[24]
			405	400	[55]	**1.01**	909^⁎^	[55]				61 (graph)	[24]	0.119	[15]	cubic [F 4 3 m]	[33]
			376	383.67	[21]	**0.98**[21]	921	[15]									
			415 (graph)	405	[24]	**1.02**											
**hex-BN**									2.2	[54]							
**CrN**												15.4+-0.5	[9]				
**HfN**	3385	[17]					341.66 - 355.46	[30]	13.9	[17]	**24.6-25.6**	15.7	[9]			fcc	[17]
	3225	[7]										17.0+-2.0	[27]				
**NbN**	2300	[9]					483.5	[9]	3.26	[7]	**60 - 148.3**					cubic	[9]
	2330	[7]					195.6	[7]									
**Si**_**3**_**N**_**4**_	1900	[13]	290	120	[13]	**2.42**	295	[34]	3.18	[13]	**66.7 - 92.8**	15.5	[18]	0.29	[34]	hex	[20]
	1900	[24]		115	[34]		220	[20]	3.2	[7]						hex	[18]
	1900	[7]							3.2-3.3	[54]							
	1900	[20]							3.2	[20]							
**TaN**	2700	[17]							14.3	[17]		23.7	[9]			cubic	[17]
	3075	[7]							13.8 / {14.36}	[9]							
**TiN**	2930	[13]	295	212.23^⁎^	[21]	**1.39****[21]**	600	[13]	5.44	[13]	**46.2 - 115.4**	21.0+-3.0	[27]			cubic	[20]
	2950	[20]	320^⁎^		[37]		260	[20]	5.4	[20]						fcc	[17]
	2950	[9]					251.1	[9]	5.43 / {5.44}	[9]							
	2950	[17]					445 - 472.06	[30]	5.39	[17]							
	2900	[7]							5.2	[7]							
**VN**	2050	[9]							6.040 / 6.102	[6]		**15.5+-1.5**	[27]				
	2200	[7]							6	[7]							
**ZrN**	2980	[20]							7.3	[20]	**50**[7]	**18.0+-2.0**	**[27]**			cubic	[20]
	2980	[9]							7.29	[17]						fcc	[17]
	3950	[17]															
	2950	[7]															
**Intermetallics**																	
**Be**_**12**_**Ti**	1593	[2]	117	128	[56]	**0.91**	282	[56]	2.3	[2]	**122.6**			0.099	[56]	D2b tI26	[56]
																P6/mmm	[2]
**CoAl**	1635	[56]	162	114	[56]	**1.42**	278	[56]				5.2	[80Sam]	0.214	[56]	B2cP2	[56]
**CoSi**_**2**_	1326	[2]	210.1	67.5	[56]	**3.11**	182.9	[56]	4.94 / {4.95}	[9]	**36.9 - 37.0**	5.4	[9]	0.355	[56]	Fm3m	[2]
	1277	[6]															
**CrSi**_**2**_	1475	[2]	172	153.3	[56]	**1.12**	354.6	[56]	4.91	[2]	**71.1 - 72.1**	6.9	[9]	0.156	[56]	P6222	[2]
	1500 +-20	[6]							4.91 / {4.978}	[9]							
**Fe**_**3**_**Al**									{6.648}	[9]							
**FeAl**							261	[56]	5.585	[9]	**46.7**					B2cP2	[56]
**MoSi**_**2**_	2050	[56]	209.7	191.1	[56]	**1.10**	384		5.9 - 6.3 / {6.24}	[6]	**60.3 - 71.5**	12.95 - 15.2	[6]	0.151	[56]	tet	[18], [56]
	2030	[20]					380	[20]	6.2	[20]		6.9	[9]			tet	[20]
	2030	[6]					421.7	[6]								I4/mmm	[2]
**Ni**_**3**_**Al**			173	77.3	[56]	**2.24**	201.9	[56]	{7.293}	[9]				0.305	[56]		[56]
**Ni**_**3**_**Fe**			180.6	85.5	[56]	**2.11**	221.4	[56]						0.296	[56]		[56]
**NiAl**	1638	[56]	166	70	[56]	**2.37**	184.1	[56]	5.85		**31.5**			0.315	[56]		[56]
**TaSi**_**2**_							338	[13]	9.1	[13]	**37.1 - 38.3**	9.8 - 11.8	[9]			P6222	[2]
									9.1	[2]							
									8.83 / {9.1}	[9]							
**Ti**_**3**_**Sn**			97.5	41.9	[56]	**2.33**	110	[56]						0.312	[56]		[56]
**TiAl**			110	70	[56]	**1.57**	173	[56]	3.84 / {3.63}	[9]	**45.1 - 47.7**	1.8	[9]	0.234	[56]	L10 tP4	[56]
**TiAl**_**3**_	1375	[56]	105.6	93	[56]	**1.14**	215.7	[56]	3.31 / {3.371}	[9]	**64.0 - 65.2**	6.7	[9]	0.16	[56]		[56], [57]
**TiCr**_**2**_	1550	[56]	159	71	[56]	**2.24**	184	[56]						0.31	[56]	C14. hP12	[56]
**TiSi**_**2**_			148.9	116.7	[56]	**1.28**	277.8	[56]	4.39 / {4.13}	[6]	**59.0 - 69.1**	6.78	[9]	0.189	[56]	Fddd	[6]
							258.9	[6]	4.02 / {4.043}	[9]							
**Ti**_**5**_**Si**_**3**_	2150	[7]							4.2	[7]							
**VSi**_**2**_			166 - 167.2	142 - 147.9	[56]	**1.12**	331 - 342.6	[56]	4.34 / {4.627}	[9]	**71.5 - 78.9**	8.7 - 9.4	[9]	0.158	[56]		[56]
**V**_**5**_**Si**_**3**_									5.1	[7]							
**WSi**_**2**_	2180	[7]	222.4	203.6	[56]	**1.09**	467.9	[56]	9.25 / [9.857]	[9]	**47.5-50.6**	10.5	[9]	0.149	[56]		[56]
									9.25	[7]							
**YAl**_**2**_			89.2	65.5	[56]		158	[56]	{3.933}	[9]	**40.2**			0.205	[56]	C15 cF24	[56]
**YFe**_**2**_			97	49.6	[56]	**1.36**	127	[56]								C15 cF24	[56]
**ZrAl**_**2**_	1645	[56]	117	93.8	[56]	**1.25**	222	[56]	4.42 / {4.561}	[9]	**48.7 - 50.2**			0.184	[56]	C14. HP12	[56]
**ZrAl**_**3**_	1580	[56]	95.4 - 106.9	85.1 - 90.0	[56]	**1.06**	201.8 - 205	[56]	4.11 / {4.098}	[9]	**49.1-50.0**	5.5	[9]	0.185	[56]		[56]
